# Sometimes

**Published:** 1867-12

**Authors:** 


### SOMETIMES, without apparent cause, a person will suddenly wake up to the knowledge that his feet are cold and a disagreeable sensation is caused which pervades the whole body and the mind and temper become fretful and morose; this is often the case in the very midst of summer; when this is observed you are taking cold and you should instantly treat the feet to a blazing fire as named above; if this is not practicable, give them a hot foot bath as just directed; in either case you will not only avert the cold but you will experience a feeling of comfortableness, which is absolutely delightful; this same kind of bath is the speediest and most comfortable means of warming the feet when they are found to be uncomfortable cold after coming in from a walk, or a long day's work.

Many ways have been devised for rendering the upper leather of shoes impervious to water; a much better plan is to keep out of the water, for whatever will keep water out will also keep the perspiration and ill odor always in; some persons' feet are smelled a mile off, more or less. To make leather impervious, is to make it board like, hard, unyielding, and hot as fire of a summer's day; but if it be absolutely necessary at any time to wear a shoe which shall exclude water, the application of castor oil or petroleum with a brush and then allow it to dry, is perhaps the most familiar, accessible and facile mode, known. If the space between the upper leather and the sole of pegged shoes is occasionally dressed with petroleum, until the pegs are saturated with the liquid, there will be no ripping.

